# Identification of Outer Membrane Vesicles as a New Vehicle Mediating Antibiotic Resistance Gene Transfer in *Campylobacter*

**DOI:** 10.1002/jev2.70195

**Published:** 2025-11-11

**Authors:** Mengyu Zhao, Min He, Xiaolong Lin, Ke Wu, Fang Yang, Xinggui Chen, Haozheng Li, Hongning Wang, Yizhi Tang

**Affiliations:** ^1^ Key Laboratory of Bio‐Resource and Eco‐Environment of Ministry of Education, College of Life Sciences Sichuan University Chengdu China; ^2^ Animal Disease Prevention and Green Development Key Laboratory of Sichuan Province, College of Life Sciences Sichuan University Chengdu China

**Keywords:** antibiotic resistance gene, campylobacter, efflux pump, horizontal gene transfer, outer membrane vesicles, plasmid

## Abstract

The emergence and worldwide dissemination of antibiotic resistance genes (ARGs) compromise antibiotic therapy and are a major public health crisis. Horizontal gene transfer (HGT) plays a major role in the spread of ARGs among bacterial pathogens. Outer membrane vesicles (OMVs), which are membrane‐bound particles and naturally released by Gram‐negative bacteria, have been reported to carry a variety of cargos such as DNA, proteins and lipids. However, it remains unknown whether OMVs mediate transfer of ARGs in *Campylobacter*, an important foodborne pathogen whose resistance to antibiotics poses a serious threat to public health. To close this knowledge gap, we determined the role of OMVs in ARG transfer. Using a non‐conjugative plasmid (pRY112), we demonstrated that OMVs successfully transferred the plasmid from *Campylobacter coli* to *Campylobacter jejuni*. Additionally, OMVs transferred chromosomally encoded florfenicol resistance from a clinical *C. coli* isolate (SH89) to *C. jejuni*. The OMV‐mediated transfer is independent of natural transformation as both DNase I treatment (for digestion of external‐free DNA) and use of a strain deficient of natural transformation as the recipient strain did not affect OMV‐mediated ARG transfer. Transmission electron microscopy revealed direct fusion between OMVs and recipient bacterial membranes, suggesting membrane fusion as the mechanism for OMV‐mediated DNA transfer. Furthermore, we showed that OMVs derived from strains expressing a functionally‐enhanced *CmeB* (FE‐*CmeB*) transiently protect florfenicol‐susceptible *C. jejuni* against selection by the antibiotic. Together, these findings indicate that OMVs mediate the transfer of both plasmid‐ and chromosome‐encoded ARGs in *Campylobacter* and define OMVs as a novel pathway for *Campylobacter* to acquire antibiotic resistance via HGT.

## 1 Introduction

Horizontal gene transfer (HGT) plays an important role in genetic exchange in bacterial communities, contributing to the exchange of genetic materials and spread of fitness‐promoting factors, such as antibiotic resistance genes (ARGs) (Nazarian et al. 2018). The well‐known mechanisms for HGT include conjugation‐mediated transfer of plasmids, phage‐mediated transduction and natural transformation that occurs in some bacterial species (Soucy et al. 2015; Brito 2021). Recently, bacterial outer membrane vesicles (OMVs), which are membrane‐derived lipid bilayers of Gram‐negative bacteria, are also proposed to be a potential pathway for genetic exchange among Gram‐negative bacteria, possibly by a fusion and release mechanism that delivers OMV‐contained DNA to recipient cells (Schwechheimer and Kuehn 2015).

Thermophilic *Campylobacter* species, particularly *Campylobacter jejuni* and *Campylobacter coli*, are a leading cause of human gastroenteritis worldwide (Facciola et al. 2017). It is estimated that 1.5 million cases of *Campylobacter* infections occur in the United States alone on an annual basis (Fischer et al. 2024). As a zoonotic pathogen, *Campylobacter* is transmitted to human through multiple routes, including raw or undercooked chicken meat, unpasteurized milk, contaminated food and water and direct contact with animals (Luangtongkum et al. 2009). Clinical treatment of human campylobacteriosis often uses fluoroquinolones (e.g., ciprofloxacin) or macrolides (e.g., azithromycin), although other classes of antibiotics may be prescribed for different conditions (Shen et al. 2018; Tang et al. 2017). However, antibiotic use in healthcare and veterinary medicine has made *Campylobacter* increasingly resistant to clinically used antibiotics (Wallace et al. 2021), limiting therapeutic efficacy and options for patients. Because of the significant impact on food safety and public health, the Centers for Disease Prevention and Control (CDC) classified antibiotic‐resistant *Campylobacter* as a serious antimicrobial resistance threat in the United States (https://www.cdc.gov/drugresistance/pdf/threats‐report/2019‐ar‐threats‐report508.pdf).

The genomic contents of *Campylobacter* vary significantly from strain to strain, and this variation facilitates *Campylobacter* adaptation to various environments (Meinersmann et al. 2002; Wang et al. 2024; Parkhill et al. 2000). *Campylobacter* generates genetic diversity by mutation and HGT (Golz and Stingl 2021). It has been known that *Campylobacter* is capable of using natural transformation, conjugation and transduction for HGT (Golz and Stingl 2021). Particularly, *Campylobacter* species are naturally competent and exhibit high efficiency in the uptake of DNA from the same species but low efficiency with DNA from different bacterial species (Vegge et al. 2012). Several genes involved in natural transformation have been identified, including *Cj1211* that encodes an inner membrane protein responsible for importing unzipped single‐stranded DNA into the cytoplasm (Golz et al. 2023; Jeon et al. 2008; Jeon and Zhang 2007). Insertional mutagenesis of *Cj1211* abolish natural transformation in *C. jejuni* (Golz et al. 2023; Jeon et al. 2008). Additionally, the RA^m6^ATTY motif was found to function as a DNA uptake sequence and significantly influences natural transformation frequencies in *C. jejuni* (Beauchamp et al. 2017). Conjugation requires physical contact between a donor and a recipient cell, through which conjugative and mobilizable plasmids are transferred (Soucy et al. 2015). The transfer of plasmids in *Campylobacter* via conjugation has been frequently reported (Tang et al. 2017; Marasini et al. 2018; Taylor et al. 1981). Bacteriophages have also been found in *Campylobacter* and contributed to the genetic diversity of *Campylobacter*. For example, *C. jejuni* RM1211 carries *C. jejuni*‐integrated elements (CJIEs) that originate from phages (Barton et al. 2007). Whole genome sequences also revealed the presence of various phages in *Campylobacter* isolates (Hooton et al. 2016), suggesting a role of transduction in HGT between *Campylobacter* strains.

OMVs have been found to mediate diverse functions, such as transferring and disseminating a variety of bacterial cargos, providing immediate protection for nearby susceptible bacteria and regulating microbial interactions within bacterial communities (Schwechheimer and Kuehn 2015; Yao et al. 2023; Fulsundar et al. 2014). OMVs, containing outer‐membrane lipids, proteins, DNA and soluble periplasmic contents, are not the products of cell lysis but are actively produced by bacteria (Schwechheimer and Kuehn 2015). Increasing evidence has shown that OMVs of Gram‐negative bacteria or membrane vesicles (MVs) of Gram‐positive bacteria play important roles in the dissemination of genetic substances, including ARGs (e.g., *bla*_CTX‐M‐15_, *bla*_NDM‐1_ and *optrA*) and virulence genes (Yao et al. 2023; Bielaszewska et al. 2020; Chatterjee et al. 2017; Yaron et al. 2000; Dell'Annunziata et al. 2021; Zhao et al. 2024). Recently, OMV‐mediated gene transfer has been recognized as the fourth mechanism for HGT among bacteria, indicating its important role in generating genetic diversity (Brito 2021; Dell'Annunziata et al. 2021). Although how exactly OMVs transfer DNA remains unclear, it has been proposed that OMVs fuses with recipient cell membranes and deliver cargos into the cells (Brito 2021; Fulsundar et al. 2014).

*Campylobacter* can naturally produce OMVs during growth, and these OMVs have been shown to deliver cargos of toxins and virulence proteins into host cells (Khan et al. 2023); however, whether OMVs mediate HGT of AMR genes/determinants between *Campylobacter* species/strains is unknown. Given the importance of HGT in facilitating *Campylobacter* adaptation and the heightened importance of antibiotic‐resistant *Campylobacter* in public health, we sought to determine the potential role of OMVs in mediating transfer of AMR genes in *Campylobacter* in this study. We demonstrated that *Campylobacter*‐derived OMVs transferred both plasmids and chromosome‐encoded ARGs into recipient *Campylobacter* cells. We also observed that OMVs loaded with drug‐binding proteins (notably efflux proteins) may provide immediate protection for antibiotic susceptible bacterial cells. To the best of our knowledge, this is the first report on OMV‐mediated ARG transfer in foodborne pathogen *Campylobacter*.

## 2 Materials and Methods

### 2.1 Bacterial Strains and Culture Conditions

*C. coli* SH89 was originally isolated from a swine slaughterhouse in Sichuan Province, China (Tang et al. 2020). It contained the *cfr*(C) gene on a plasmid and showed resistance to florfenicol (64 mg/L), erythromycin (32 mg/L), tetracycline (>128 mg/L) and ciprofloxacin (64 mg/L). *C. jejuni* NCTC 11168 was used as the recipient strain for HGT and generation of mutant constructs. *C. coli* 33559 was used for transformation with pRY112 (Yao et al. 1993). *Campylobacter* isolates were cultured using Mueller–Hinton (MH) broth or agar at 42°C under microaerobic conditions (5% O_2_, 10% CO_2_ and 85% N_2_). *Escherichia coli* was grown in LB broth at 37°C for 24 h under aerobic conditions. When needed, the culture media were supplemented with chloramphenicol (20 mg/L), florfenicol (4 mg/L), or kanamycin (50 mg/L).

### 2.2 Construction of the Cj1211 Mutant in *C. jejuni* NCTC 11168

The *Cj1211* gene in *C. jejuni* encodes the inner membrane channel protein responsible for importing single‐stranded DNA into the cytoplasm and therefore plays a critical role in the natural transformation of *C. jejuni* (Golz et al. 2023; Jeon et al. 2008). Previously it was shown that mutation of *Cj1211* abolished natural transformation in *Campylobacter* (Golz et al. 2023; Jeon et al. 2008). To exclude the possibility of natural transformation in OMV‐mediated DNA transfer, we generated a *Cj1211* mutant by inserting *aphA3*, encoding a kanamycin resistance gene, into *Cj1211*. Briefly, primers *Cj1211*‐5F and *Cj1211*‐5R were used to amplify the 5′ fragment of *Cj1211* and its upstream region (*Cj1211*‐5′ fragment), while primers *Cj1211*‐3F and *Cj1211*‐3R were used to amplify the 3′ region of *Cj1211* and its downstream region (*Cj1211*‐3′ fragment) (Table S1). The primer pair *aphA3*‐F/*aphA3*‐R was used to amplify the *aphA3* gene from the pRRK plasmid using Phusion High‐Fidelity DNA Polymerase (Thermo Fisher Scientific) (Karlyshev and Wren 2005). After purification, the *Cj1211*‐5′, *aphA3* and *Cj1211*‐3′ PCR fragments were ligated by Gibson Assembly and amplified using the *Cj1211*‐5F and *Cj1211*‐3R primers, resulting in the construction of the *Cj1211*‐5′‐*aphA3*‐*Cj1211*‐3′ PCR product. The purified *Cj1211*‐5′‐*aphA3*‐*Cj1211*‐3′ product was then electroporated into competent *C. jejuni* NCTC 11168 cells. Transformants were selected on MH agar plates supplemented with 50 mg/L kanamycin. The *Cj1211* mutant was confirmed by PCR and was designated YZ2024.

### 2.3 Transfer of the pRY112 Plasmid Into *C. coli*

pRY112 is a *Campylobacter* shuttle vector that is a 6.45 kb plasmid carrying *Campylobacter* and *E. coli* replicons, as well as the chloramphenicol resistance‐encoding gene *Cm^R^*. To examine whether *Campylobacter*‐OMVs could mediate plasmid transfer between *C. coli* and *C. jejuni*, pRY112 purified from *E. coli* was electroporated into *C. coli* 33559, and the resulting *C. coli* carrying pRY112 was designated *C. coli* CP112 in this study.

### 2.4 Antimicrobial Susceptibility Testing

Antimicrobial susceptibility testing was conducted by the standard microtiter broth dilution method according to the guidelines of the Clinical and Laboratory Standards Institute (CLSI 2016). *C. jejuni* ATCC 33560 was used as the quality control.

### 2.5 OMVs Isolation and Characterization

*C. coli* strains CP112 and SH89 were inoculated into 3 L of MH broth and incubated at 42°C to an OD_600_ of ∼1.0 in jars filled with premixed gas (5% O_2_, 10% CO_2_ and 85% N_2_). The cultures were centrifuged at 12,000 × *g* at 4°C for 15 min and supernatants were collected. This centrifugation step was repeated 2–3 times to remove intact bacterial cells. The supernatants were sterile‐filtered with 0.45 µm Stericup filters (Millipore Corporation, USA) to remove residual bacteria and concentrated with Vivaflow 50 (Sartorius, Germany). Vesicles were collected by ultracentrifugation at 140,000 × *g* at 4°C for 2 h and resuspended in 2 mL of PBS (crude OMVs). The suspension was further purified by glycerol density gradient centrifugation (DGC) at 140, 000 × *g* at 4°C for 2.5 h. The precipitate was resuspended in PBS, and then the suspension was further sterile‐filtered with 0.22 µm Stericup filters (Millipore Corporation, USA) to remove any remaining bacteria. To check for the absence of bacterial contamination, the filtered OMVs were plated on MH agar plates and cultured at 42°C for 2 days under microaerobic conditions. Additionally, an aliquot of filtered OMVs were treated with 70 U/mL DNase I to remove extravesicular DNA, and reactions were stopped by heat treatment of the mixtures for 15 min at 65°C. The morphology and size of OMVs produced by *C. coli* strains were visualized by transmission electron microscopy (TEM). OMV samples were placed on copper grids and negatively stained with 2.0% (w/v) phosphotungstic acid at room temperature for 90 s. The OMVs were then observed via TEM (Hitachi, HT‐7700, Japan). The concentration and size distribution of the OMVs were measured using a nanoparticle tracking analysis (NTA) instrument (Zeta View PMX 110) and its corresponding software (version 8.05.14 SP7) (Zhao et al. 2024).

### 2.6 Internal OMV‐Associated DNA Extraction and Sequencing

The purified OMVs were further treated with DNase I to ensure complete digestion of external OMV‐associated DNA, followed by extraction of the internal DNA using a DNA extraction kit. The concentration and purity of the extracted DNA were measured with a Qubit4 fluorometer. The DNA samples were sequenced on an Illumina TruSeq platform (Shanghai Personal Biotechnology Co., Ltd., China) using a 350‐bp paired‐end library with ∼200‐fold average coverage. The paired‐end reads were assembled de novo using SOAPdenovo v2.04 and Gapcloser v1.12. MCScanX was used to align the OMV DNA against the whole‐genome sequence of the donor strain *C. coli* SH89.

### 2.7 OMV‐Mediated Transfer of Antibiotic Resistance Determinants

OMV‐mediated transformation was conducted following a previously described protocol with minor modifications (Zhao et al. 2024). Briefly, recipient *C. jejuni* strains YZ2024 and wide‐type NCTC 11168 were cultured in MH broth and then centrifuged to collect the pellet which was then resuspended using fresh MH to 10^8^ cfu/mL. For transformation assays, 100 µL of recipient cell suspension (1 × 10^7^ CFU) was mixed with 20 µL of either CP112‐OMVs or SH89‐OMVs containing approximately 50 ng DNA. The mixture was adjusted to 10 mL with fresh MH broth, then supplemented with 125 µL DNase I (0.55 U/µL; Sigma–Aldrich). The transformation cultures were incubated under microaerobic conditions (85% N_2_, 10% CO_2_, 5% O_2_) at 42°C with shaking (180 rpm) for 16 h. The bacteria were centrifuged, and the pellet was resuspended in 1 mL of PBS. A 100 µL aliquot of the suspension was plated onto MH agar plates supplemented with 4 mg/L florfenicol and incubated for 48 h. Control experiments were conducted by mixing 50 ng of plasmid or genomic DNA extracted from CP112 and SH89 with recipient strain YZ2024 under identical conditions. Additionally, to assess whether structural integrity of OMVs was required for DNA transfer, OMVs purified from CP112 and SH89 were disrupted using 2% Triton X‐100 prior to incubation with the recipient strains under the same conditions described above. For all transformation experiments, the transformation frequency was defined as the number of vesiculants per 1 µg of DNA divided by the total number of recipient cells. Each experiment was performed in triplicate

### 2.8 OMV‐Mediated Plasmid Transfer in Broth Culture

Fresh cultures of the donor strain CP112 (Cm^R^) and the recipient strain YZ2024 (Kan^R^) were adjusted to ∼1.0 × 10^7^ cfu/mL. Of each culture, 100 µL was inoculated into 4 mL of MH broth, and the mixture was incubated at 42°C with shaking (180 rpm) under microaerobic conditions for 18 h. The co‐culture was centrifuged, and the pellet was resuspended in 500 µL of MH broth, from which 100 µL was spread onto a MH agar plate supplemented with 32 mg/L kanamycin and 4 mg/L chloramphenicol. Vesiculants were selected from the plate and the presence of both *aphA3* and *cat* were determined by PCR.

### 2.9 Membrane Fusion Assay

To examine whether OMVs interact with the membrane of recipient cells, 20 µL of *C. jejuni* YZ2024 culture (OD_600_ ≈ 0.8) was mixed with 100 µL CP112‐OMVs, and the mixture was incubated at 42°C for 4 h under microaerobic conditions with shaking at 120 rpm. Following incubation, the bacterial cells were examined by TEM (JEOL Ltd. JEM‐2100Pluzs) to observe OMV‐bacterial cell interaction.

### 2.10 WGS and Analysis

DNA libraries of 16 SH89‐vesiculants (11168‐01 to 11168‐16) were constructed and then sequenced on an Illumina MiSeq platform (Shanghai Personal Biotechnology Co., Ltd., China) using a 400‐bp paired‐end library with ∼200‐fold average coverage. After the adaptor sequence and quality bases were removed, the resulting reads were subsequently de novo assembled into contigs, which were subsequently aligned against the reference genome *C. jejuni* NCTC 11168 using Mauve (v 2.3.1). The contigs were aligned against NCTC 11168 using an online tool (http://genome.cs.nthu.edu.tw/Multi‐CSAR/).

### 2.11 Natural Transformation

Natural transformation was performed according to methods described previously (Wang and Taylor 1990). Briefly, purified plasmid pRY112 from *C. coli* 33559 and genomic DNA extracted from *C. coli* (SH89‐DNA) were used as donor DNA, and the *C. jejuni* strains NCTC 11168 and YZ2024 were used as the recipient strains. Transformants were selected on MH agar plates supplemented with 20 mg/L chloramphenicol or 4 mg/L florfenicol. The transformation frequency was defined as the number of transformants divided by the total bacterial number per 1 µg of DNA. Each transformation experiment was repeated three times.

### 2.12 In Vitro Assay of the Protective Effect of OMVs on Antibiotic‐Susceptible Campylobacter

OMVs isolated from NCTC 11168, 11168‐FE*cmeRAB*, which is a derivative of NCTC 11168 transformed with the FE‐*cmeRAB* of strain SH89 (Lin et al. 2025), and SH89 were used for antimicrobial susceptibility assays. The antibiotic susceptible strains 11168 and YZ2024 were cultured to an OD_600_ of 0.8. Bacterial cells were harvested by centrifugation (6000 × *g* for 10 min), resuspended in MH broth and adjusted to approximately 1 × 10^8^ cfu/mL. Each suspension was mixed with an equal volume of OMVs (1 × 10^12^ particles/mL in MH broth). Florfenicol and ciprofloxacin were diluted with MH broth in a 96‐well plate, and to each well was added with 100 µL of the cell‐OMV mixture. The plate was incubated at 42°C for 24 h, and the MICs were evaluated according to CLSI guidelines. Wells without added OMVs were used as control. All experiments were performed in triplicate.

## 3 Results

### 3.1 Morphological Characterization of OMVs

**FIGURE 1:**
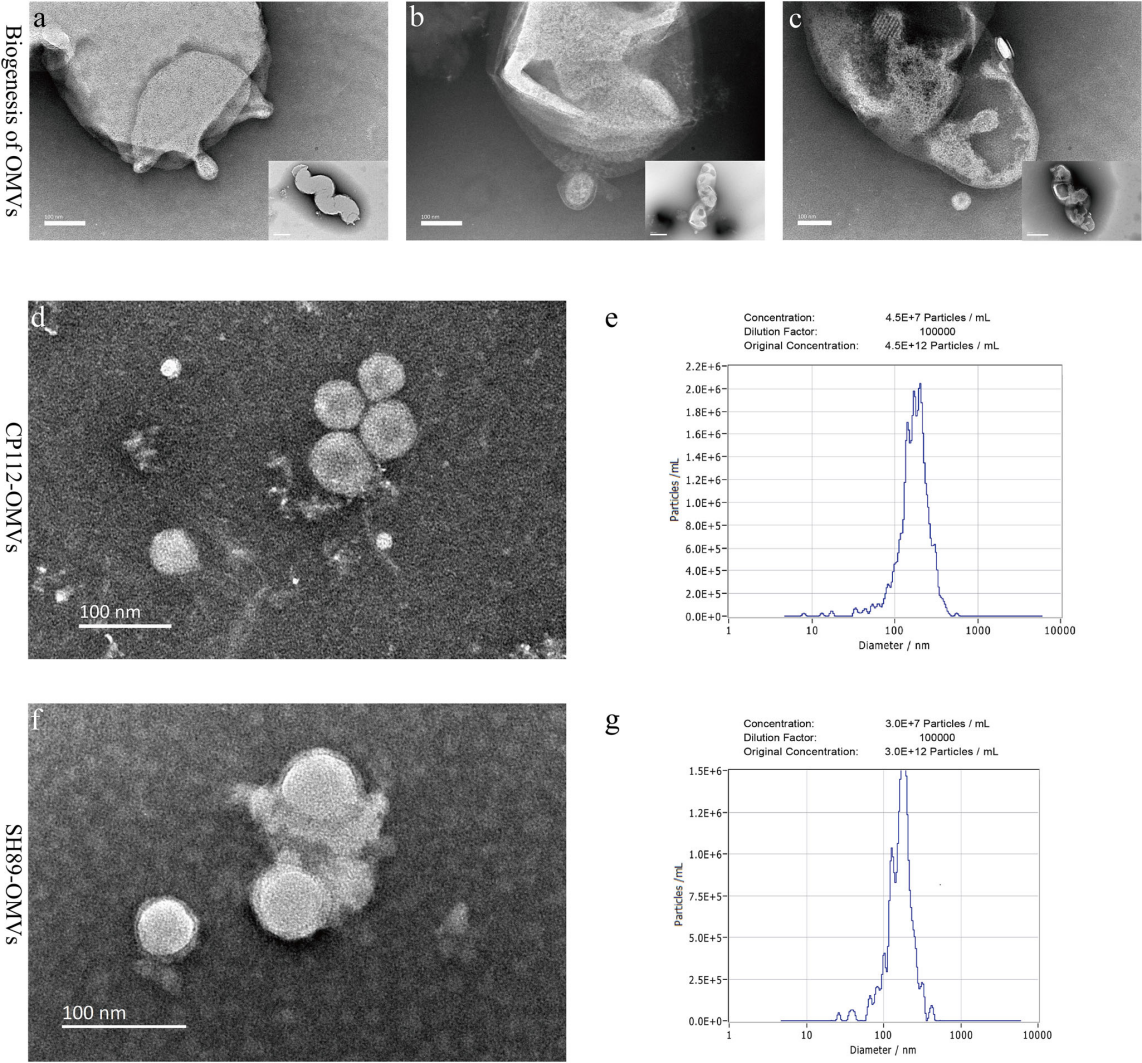
Characterization of OMVs produced by *C. coli* CP112 and SH89. (a–c) Biogenesis of CP112‐OMVs: (a) protrusion, (b) vesiculation and (c) release. (d, f) CP112‐ and SH89‐OMVs observed by TEM. The scale bar indicates 100 nm. (e, g) The size distribution of CP112 and SH89 OMVs detected by NTA.

The biogenesis of *Campylobacter*‐derived OMVs was visualized using TEM, revealing three distinct stages: membrane protrusion (Figure 1a), OMV formation (Figure 1b) and OMV release (Figure 1c). These observations suggest that *Campylobacter* secretes OMVs primarily through a process resembling exocytosis‐like membrane blebbing. OMVs were purified from *C. coli* CP112 and SH89, which are *C. coli* 33559 containing pRY112 and a clinical *C. coli* isolate, respectively (see Materials and Methods). TEM revealed that these purified OMVs were electron‐dense particles of varying sizes and were spherical in shape with intact bilayer membranes (Figure 1d,f). NTA revealed that the majority of CP112‐OMVs ranged in size from 99 and 243 nm, and the mean diameter was 172.9 ± 62 nm (Figure 1e). Similarly, SH89‐OMVs ranged from 100 to 267 nm, with a mean diameter of 182.7 ± 69.3 nm (Figure 1g). The concentrations of CP112‐OMVs and SH89‐OMVs were approximately 4.5 × 10^12^ and 3.0 × 10^12^ particles/mL, respectively (Figure 1e,g). There were no visible bacteria under the electron microscope, and no bacterial growth was observed on the culture plate suitable for *Campylobacter* growth, indicating that the OMVs were free of whole bacterial cells.

### 3.2 DNA Content Characterization of OMVs

**FIGURE 2:**
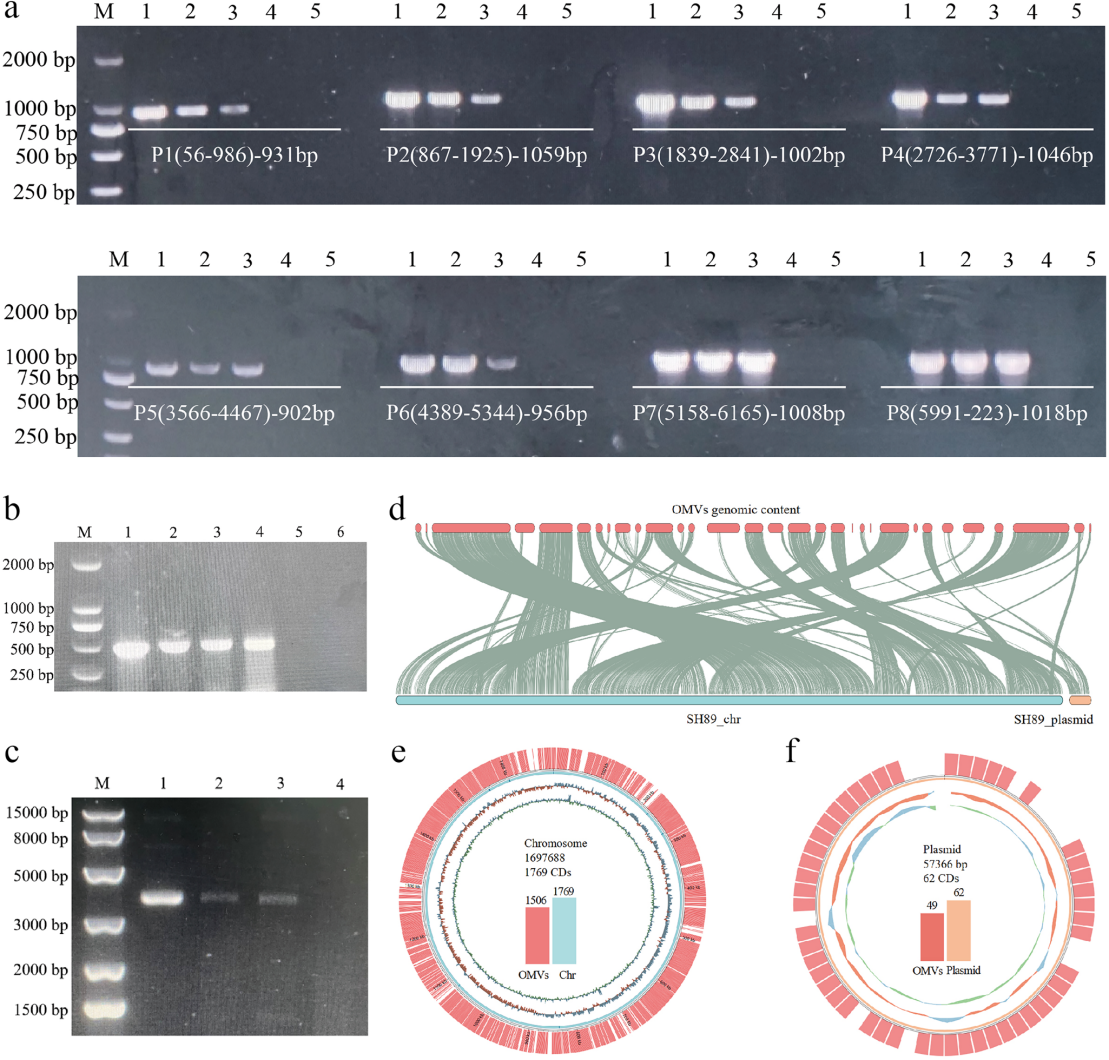
DNA content analysis of *Campylobacter* OMVs. (a) Detection of plasmid pRY112 in CP112‐OMVs by PCR. Eight primer pairs spanning the entire plasmid pRY112 were used to amplify DNA from DNase I‐treated OMVs. Primer positions and expected product lengths are labelled on each gel image. Lane M, DNA ladder; lane 1, plasmid pRY112 extracted from CP112 as template (positive control); lane 2, DNA extracted from CP112‐OMVs as template; lane 3, DNA extracted from DNase I‐treated CP112‐OMVs (external DNA removed) as template; lane 4, DNase I‐treated free plasmid pRY112 as template (degraded control); lane 5, negative control (no template). (b) PCR verification of the Cm^R^ gene in CP112 OMVs. Lane 1, purified plasmid pRY112 as template (positive control); lane 2, genomic DNA from CP112 as template; lane 3, DNA from CP112‐OMVs as template; lane 4, DNA from a CP112‐vesiculant as template; lane 5, DNA from recipient strain YZ2024 as template; lane 6, negative control (no template). (c) Electrophoresis of plasmid pRY112 extracted from *E. coli DH5α* carrying pRY112 (Lane 1), *C. coli* CP112 (Lane 2), CP112‐visiculants (Lane 3) and the recipient strain YZ2024 (Lane 4; negative control). (d) Collinearity analysis of SH89‐OMV genomic DNA content. MCScanX alignment of OMV‐derived DNA sequences (red) against the *C. coli* SH89 chromosome (blue) and endogenous plasmid pSH89 (yellow). (e) Coverage of SH89 chromosomal DNA in OMVs. OMV‐derived sequences mapped to 85.13% (1506/1769 CDs) of the SH89 chromosome. (f) Coverage of plasmid pSH89 in OMVs. OMV‐derived sequences encompassed 79.01% (49/62 CDs) of the plasmid.

DNase I was used to treat CP112‐OMVs and SH89‐OMVs to remove external DNA. Due to the low plasmid content within OMVs, conventional plasmid extraction and electrophoresis proved insufficient to confirm plasmid presence. To address this, DNase I‐treated CP112‐OMVs were lysed to release internal DNA, and eight primer pairs spanning the entire plasmid pRY112 were used for PCR amplification. This approach successfully demonstrated the presence of intact pRY112 in CP112‐OMVs (Figure 2a). Subsequent PCR analysis further confirmed that CP112‐OMVs harboured the chloramphenicol resistance gene (Cm^R^), a key marker of plasmid pRY112 (Figure 2b). Additionally, the presence of plasmid pRY112 in donor strain CP112 was also demonstrated by extracting plasmids and confirming their presence through electrophoresis (Figure 2c). Collectively, these results provide robust evidence that CP112‐OMVs carry plasmid pRY112.

To analyse the genomic content of SH89‐OMVs, purified vesicles (∼3 × 10^12^ particles) were treated with DNase I to remove external DNA. Internal DNA isolated from DNase I‐treated OMVs was subjected to next‐generation sequencing (NGS). Collinearity analysis revealed that OMV‐derived DNA sequences aligned to the SH89 chromosome spanned 1506 coding sequences (CDs), covering 85.13% (1506/1769 CDs) of the whole genome (Figure 2d,e). Additionally, SH89 carries a 57.366 kb endogenous plasmid, and OMV‐derived DNA mapped to the plasmid encompassed 49 CDs, representing 79.01% (49/62 CDs) of the plasmid sequence (Figure 2d,f). To assess potential gene enrichment within OMVs, we analysed sequence read distribution across the genome. The data showed near uniform coverage without localized enrichment, indicating that SH89 DNA is randomly packaged into OMVs rather than selectively retained. This observation suggests a passive or non‐specific incorporation mechanism during OMV biogenesis.

### 3.3 OMV Mediated the Transfer of Plasmid pRY112

As previously reported (Yao et al. 1993; Karlyshev and Wren 2005), we also showed that the shuttle plasmid pRY112 was unable to be introduced into *C. jejuni* NCTC 11168 through natural transformation in three repeated experiments (Table 1). Since CP112‐OMVs contained pRY112, we then evaluated whether OMVs can mediated plasmid transfer in *Campylobacter*. Given that *Campylobacter* is naturally transformable by free DNA, it was necessary to remove all exogenous DNA in the OMVs to eliminate the action of natural transformation. Thus, CP112‐OMVs were digested with DNase I. The purified plasmid DNA of CP112 was used as a control to verify efficiency of DNase I treatment. We first used wild‐type *C. jejuni* NCTC 11168 as the recipient for the DNA transfer experiment by using chloramphenicol as a selection marker. DNase I‐treated CP112‐OMVs yielded vesiculants at a frequency of (2.2 ± 0.76) × 10^−5^, while transformation using purified pRY112 in the presence of DNase I yielded no transformants (Table 1), indicating the effectiveness of DNase I in removing free DNA. To formally prove the role of OMVs and exclude the involvement of natural transformation in generating vesiculants, we constructed a *Cj1211* deletion mutant strain in NCTC 11168, generating a transformation‐deficient strain named YZ2024 in this study. *Cj1211* encodes a *comEC* homolog and was shown to be essential for natural transformation previously (Golz et al. 2023; Jeon et al. 2008). The natural transformation frequencies were (6.6 ± 1.58) × 10^−6^ when SH89‐DNA was introduced into NCTC 11168, while the same donor DNA yielded no transformant in YZ2024 in three repeated experiments (Table 1), indicating mutation of *Cj1211* abolished natural transformation in *C. jejuni*. Then, DNase I‐treated CP112‐OMVs and purified CP112‐DNA were mixed separately with *C. jejuni* YZ2024. CP112‐OMVs generated vesiculants at a frequency of (9.2 ± 1.05) × 10^−6^ (Table 1), while the purified pRY112 or lysed OMVs failed to produce any transformants in YZ2024. The result from YZ2024 further verified that generation of vesiculants by CP112‐OMVs is independent of natural transformation. The presence of pRY112 in the vesiculants was confirmed by both plasmid extraction and PCR amplification of the *Cm^R^* gene (Figure 2d, e). These data strongly indicated that OMVs mediate plasmid transfer in *Campylobacter*.

**TABLE 1:** Transformation frequency (mean ± SD^a^) with OMVs or purified DNA.

Recipient strains	Without DNase I treatment		With DNase I treatment			
	pRY112^b^	SH89‐DNA	SH89‐DNA	Lysed CP112/SH89‐OMVs	CP112‐OMVs	SH89‐OMVs
NCTC 11168	0	(6.6 ± 1.6) × 10^−6^	0	0	(2.2 ± 0.8) × 10^−5^	(4.6 ± 6.2) × 10^−5^
YZ2024^c^	0	0	0	0	(9.2 ± 1.0) × 10^−6^	(2.2 ± 3.1) × 10^−5^

^a^ Each frequency represents the means ± standard deviations of data from triplicate transformations from a single experiment.

^b^ Plasmid pRY112 purified from CP112.

^c^ Conferring kanamycin resistance; zero represents no transformants obtained.

### 3.4 OMVs Mediate the Transfer of Chromosome‐Encoded ARGs

**FIGURE 3:**
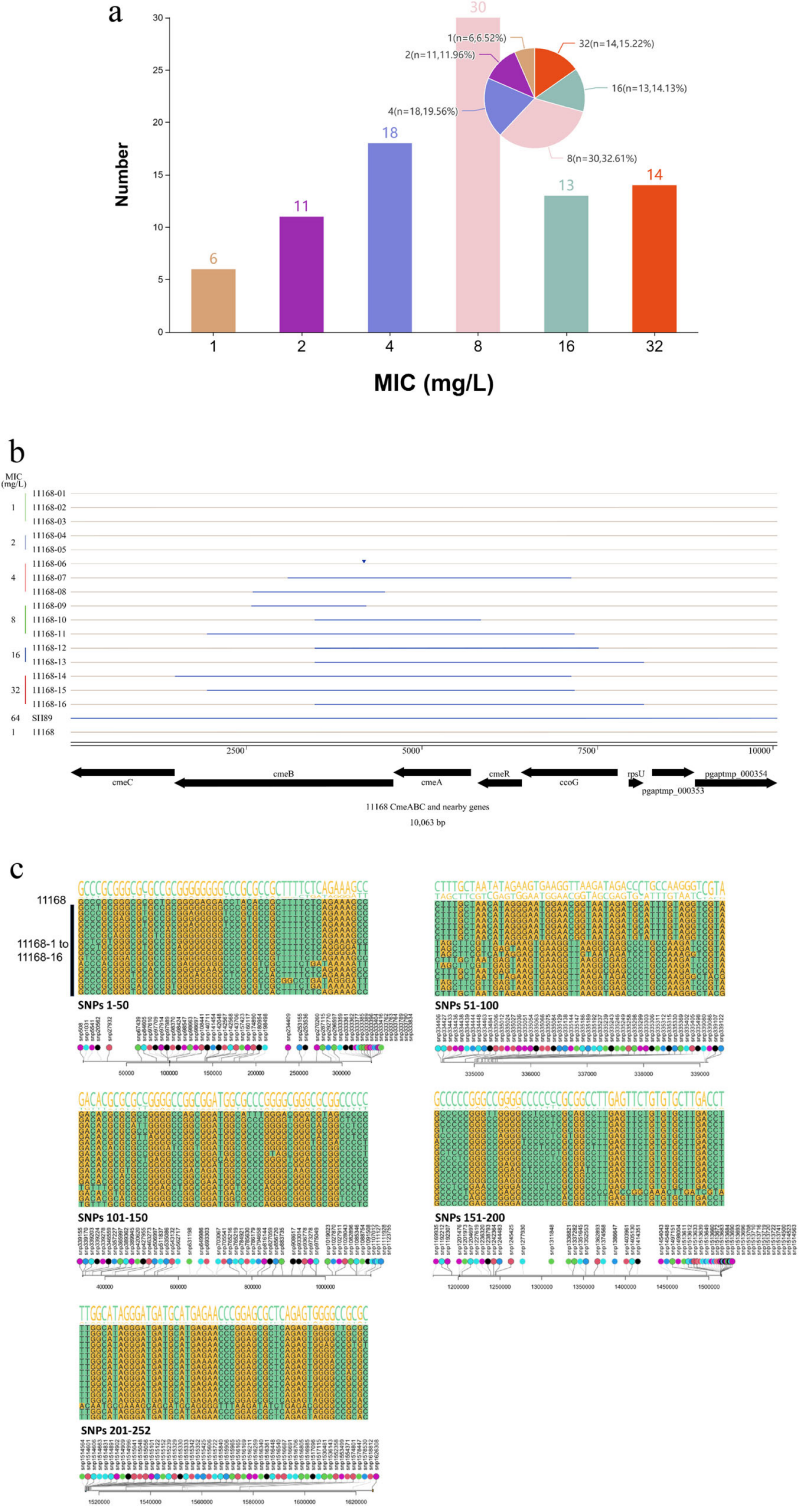
Phenotypic and genomic characterization of vesiculants derived from *C. coli* SH89‐OMVs. (a) Florfenicol MIC distribution of the 92 selected SH89‐vesiculants. (b) Sequence alignment of the *cmeABC* region from 16 vesiculants against the recipient strain NCTC 11168. Horizontal lines represent gene sequences, with brown corresponding to NCTC 11168 and blue to SH89. Blue segments within brown lines denote transferred sequence fragments from SH89 to NCTC 11168. (c) Genome‐wide distribution of 252 genetic variations identified among the 16 SH89‐derived vesiculants. Coloured dots represent individual SNPs. In the sequence logo (lower panel), nucleotide height corresponds to mutation frequency at each position. The NCTC 11168 genome serves as the reference sequence.

To investigate whether OMVs mediate the transfer of antibiotic resistance determinants encoded on chromosome, we then used OMVs purified from a natural isolate, SH89, for the transfer experiment. SH89 was a *C. coli* strain of swine origin and was resistant to multiple classes of antibiotics, including tetracycline, ciprofloxacin, florfenicol and clindamycin. Particularly, this isolate exhibited a florfenicol MIC of 64 mg/L. Therefore, we analysed the transferability of florfenicol resistance from SH89‐OMVs to *C. jejuni* NCTC 11168, which had a florfenicol MIC of 1 mg/L. SH89‐vesiculants were grown on plates containing 4 mg/L florfenicol, and the transfer frequency was found to be (4.6 ± 6.22) × 10^−5^, while no colonies were observed when DNase I treated SH89‐DNA used as donor DNA (Table 1). Furthermore, the transfer frequency was approximately (2.2 ± 3.12) × 10^−5^ when SH89‐OMVs was introduced into YZ2024 (Table 1), indicating the OMV‐mediated transfer was independent of natural transformation. In total, 92 SH89‐vesiculants were selected and analysed for their susceptibility to florfenicol, and their MIC distributions are shown in Figure 3a. Using MIC ≥ 8 mg/L as the breakpoint (CLSI 2017), a total of 57 SH89 vesiculants were resistant to florfenicol, including 14 with MICs of 32 mg/L, 13 with MICs of 16 mg/L and 30 with MICs of 8 mg/L. Interestingly, 35 SH89 vesiculants had florfenicol MICs ≤4 mg/L, although they were selected by using 4 mg/L florfenicol on plates. These included 18 vesiculants with MICs of 4 mg/L, 11 with MICs of 2 mg/L florfenicol and 6 with MICs of 1 mg/L (Figure 3a). PCR screening using *cfr*(C)‐specific primers showed that none of the 92 vesiculants contained the *cfr*(C) gene, which is located on plasmid pSH89 and is known to confer resistance to florfenicol (Tang et al. 2020). This result suggests that pSH89 was not transferred to the vesiculants.

To analyse the genetic changes associated with the florfenicol MIC increase transferred by OMVs, 2 to 3 SH89‐vesiculants in each MIC category were subjected to whole‐genome sequencing, and a total of 16 SH89‐vesiculants were sequenced, named 11168‐01 to 11168‐16 according to the ascending order of their florfenicol MICs. Multiple sequence alignment and WebLogo analysis showed that all the sequenced vesiculants were derivatives of NCTC 11168, but with single nucleotide polymorphisms (SNPs), insertions and deletions. Comparative analysis of the draft genomes of florfenicol‐resistant SH89 vesiculants with the complete genome of NCTC 11168 revealed that segments of the SH89‐derived *cmeABC* operon, especially *cmeB* gene, were transferred to the same locus in NCTC 11168 in all florfenicol‐resistant vesiculants that showed a florfenicol MIC of ≥4 mg/L (11168‐07 and 11168‐16) (Figure 3b). Vesiculant 11168‐06 had a single point mutation in the *cmeB* gene and exhibited florfenicol MIC of 4 mg/L (Figure 3b). The number of mutations in each vesiculant are listed in Table S1. A total of 252 transferred sequences were detected in the 16 vesiculants, and the sequence logo showed that they were concentrated in the region encoding the multidrug efflux operon *cmeABC* (nucleotide 331123 to 336819) (Figure 3c). Vesiculant 11168‐14 was an outlier, as it alone contained 128 transfers. In addition to the transfers in *cmeABC*, 11168‐14 also harboured transfers in two extra genes, one encoding an oligopeptide‐binding protein and the other encoding a hypothetical protein (Figure 3c).

In a previous study, we demonstrated that the *cmeB* gene of *C. coli* SH89 harboured unique mutations that along with a mutation in the *cmeABC* promoter region significantly elevated the MICs of florfenicol and other antibiotics (manuscript under minor revision). This functionally enhanced *cmeABC* was named FE‐*cmeABC* (Functionally Enhanced *cmeABC*) and carried two amino acid changes (I136 and M292) in the *CmeB* gene, which were shown to be responsible for the enhanced antibiotic resistance. Analysis of the *CmeB* sequences in vesiculants 11168‐07 to 11168‐16 revealed all of them harboured the known resistance‐conferring mutations that were transferred from SH89. This finding indicated that OMVs mediated the transfer of a chromosomally encoded FE‐*cmeB* from SH89 to NCTC 11168, which elevated florfenicol MICs in vesiculants 11168‐07 to 11168‐16.

**FIGURE 4:**
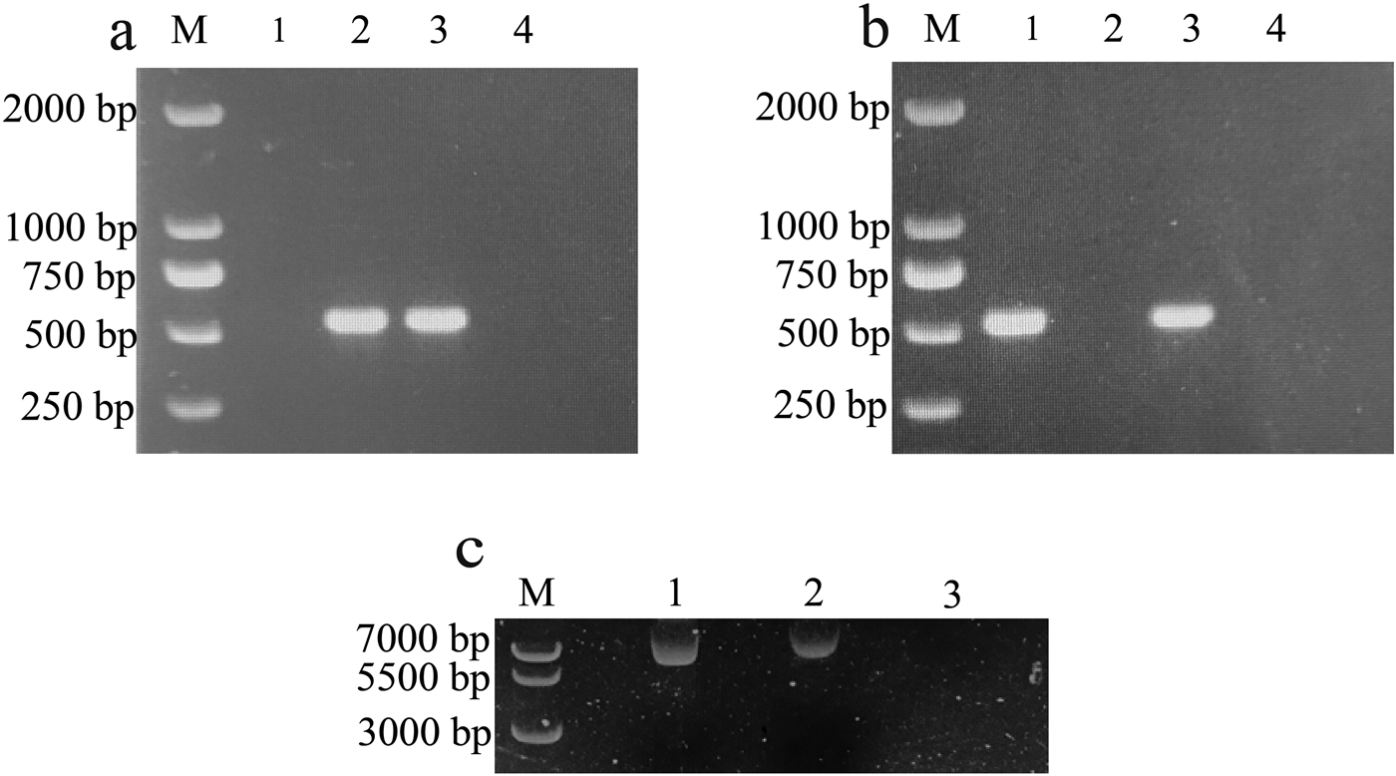
PCR confirmation of pRY112 transfer from CP112 to YZ2024 in a co‐culture experiment. Agarose electrophoresis of PCR products amplified with primers specific for *cat* (a) and *aphA3* (b) in a vesiculant. In both panels, M: DNA marker; lane 1: recipient strain YZ2024; lane 2: donor strain CP112; lane 3: vesiculant; lane 4: nuclease‐free water as negative control. (c) Electrophoresis of plasmid pRY112 extracted from *C. coli* CP112 (Lane 1), visiculants (Lane 2) and the recipient strain YZ2024 (Lane 3; no‐plasmid control).

To further investigate the potential of OMVs in mediating ARG transfer, co‐culture experiments were performed using *Campylobacter* strain CP112 as the donor and YZ2024 as the recipient. Vesiculants were obtained with a culture plate containing both kanamycin and chloramphenicol. Plasmid extraction and PCR analysis of the vesiculants confirmed the successful acquisition of the non‐conjugative plasmid pRY112 by YZ2024 (Figure 4a,b), further demonstrating that OMVs can facilitate horizontal transfer of antibiotic resistance determinants in the absence of natural transformation.

### 3.5 OMV Fusion With Bacterial Cell Membrane

**FIGURE 5:**
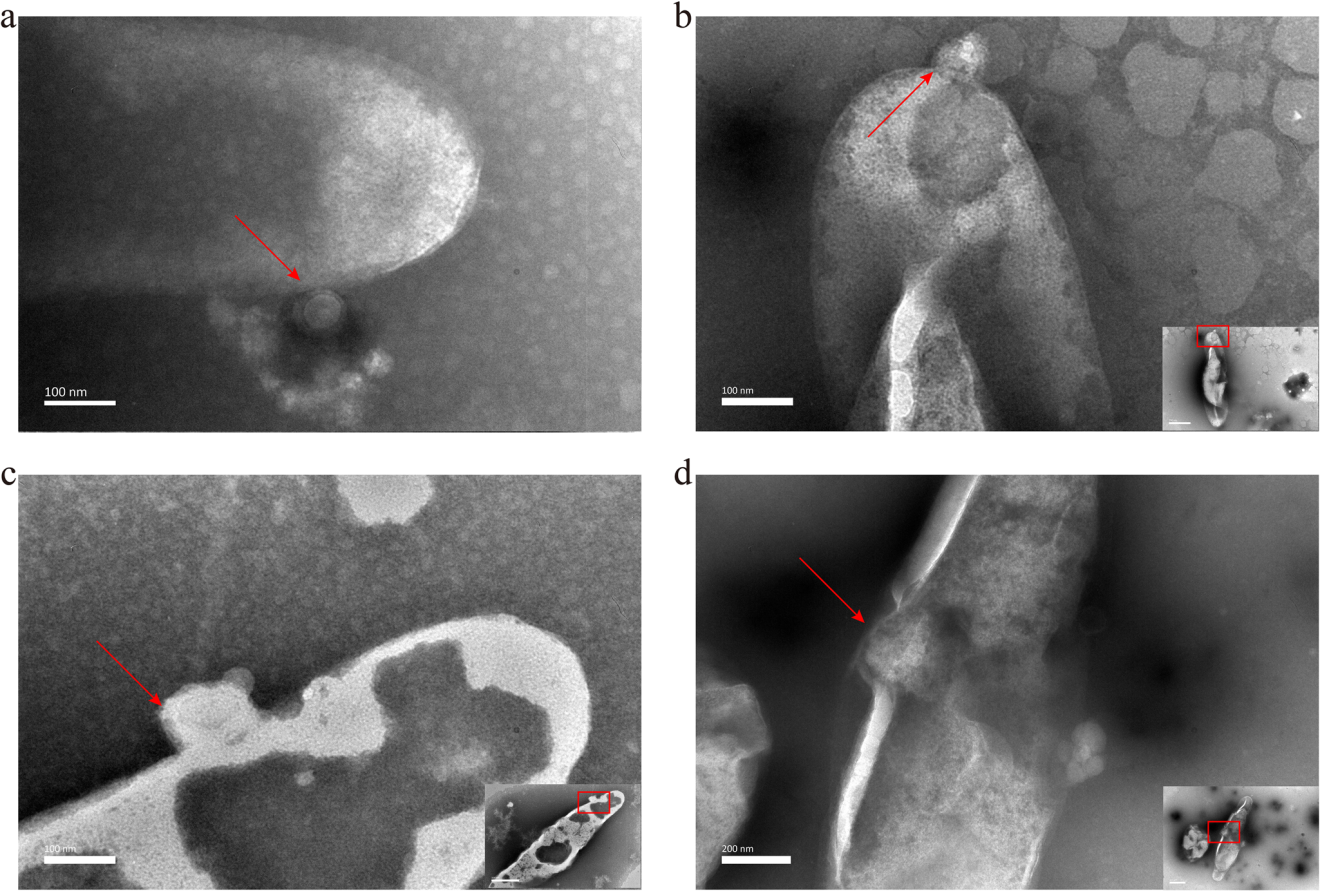
OMV fusion with bacterial membranes revealed by TEM. (a–d) TEM images showing different stages of the fusion between OMVs and the bacterial membrane (indicated by arrows), from initial contact (a), partial fusion (b), substantial fusion (c), to complete fusion (d). The inset at the bottom‐right corner of panels b, c and d shows the full view of the bacterium and OMVs (labelled with a red box).

To examine the interaction of OMVs with bacterial cells and aid understanding of the transfer mechanism, *C. jejuni* YZ2024 incubated with CP112 OMVs were observed by TEM, which captured multiple instances of OMVs fused with the membrane of recipient bacteria (Figure 5a‐d), showing morphological features highly consistent with membrane fusion events. This ultrastructural result provided direct visual evidence suggesting that DNA transfer is likely mediated via membrane fusion between OMVs and the recipient bacterial cell envelopes.

### 3.6 Transient Protection of Susceptible *C. jejuni* by OMVs Containing FE‐CmeABC

**FIGURE 6:**
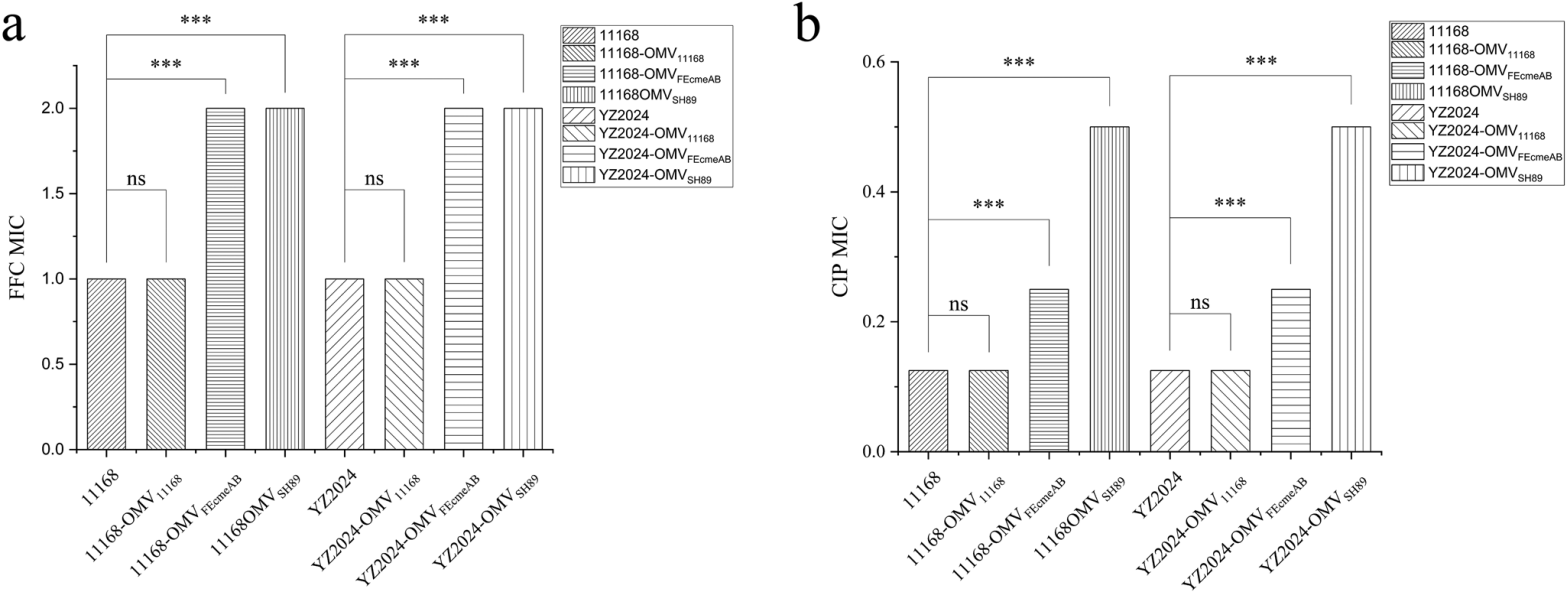
Effect of OMVs derived from various strains on the MICs of florfenicol (a) and ciprofloxacin (b) in *Campylobacter jejuni* NCTC 11168 and YZ2024. OMVs were isolated from wild‐type NCTC 11168 (11168‐OMV11168), NCTC 11168 expressing FE‐*cmeRAB* (11168‐OMVFEcmeRAB) and SH89 (11168‐OMVSH89). Data represent mean ± SD from three biological replicates. The MIC for each strain is shown without error bars as no variation between independent biological replicates was observed. Statistical significance was determined by unpaired Student's *t*‐test (ns: not significant, *p* > 0.05; ***p < 0.01).

Notably, among the sequenced vesiculants, five with florfenicol MICs of 1–2 mg/L exhibited identical *cmeABC* sequences to the recipient strain NCTC 11168, although the vesiculants with a florfenicol MIC of 2 mg/L harboured additional changes in the *porA* gene encoding the outer membrane protein. This suggests that these vesiculants with lower florfenicol MICs arose through a transient mechanism independent of *cmeABC* mutations. One possibility was that the OMVs carried antibiotic‐quenching molecules such as enzymes and CmeB proteins that compete for antibiotics, allowing the vesiculants to temporarily resist the killing effect of antibiotics. To examine this possibility, we naturally transformed SH89‐derived *cmeRAB* gene fragment into NCTC 11168. The transformant was designated as 11168‐FE*cmeRAB*. OMVs were then isolated from NCTC11168 (OMV‐11168), 11168‐FE*cmeRAB* (OMV‐FE*cmeRAB*) and SH89 (OMV‐SH89). The florfenicol MICs of 11168 and YZ2024 were measured with/without adding OMV‐11168, OMV‐FE*cmeRAB*, or OMV‐SH89. The results demonstrated that exposure of strains 11168 and YZ2024 to OMV‐FE*cmeRAB* or OMV‐SH89 resulted in a 2‐fold elevation in florfenicol MIC compared to OMV‐11168 and the untreated controls (Figure 6a). Similarly, ciprofloxacin susceptibility testing revealed a 2–4‐fold increase in MIC values when OMV‐FE*cmeRAB* or OMV‐SH89 was added to the MIC assay (Figure 6b). These findings indicate that OMVs transiently enhance antibiotic resistance by providing protective effects to susceptible bacterial strains.

## 4 Discussion

OMVs carry bacteria‐derived proteins, lipids, nucleic acids and other substances and are increasingly recognized as vital vehicles for cargo transfer and transmission (Brito 2021; Schwechheimer and Kuehn 2015). In this study, we demonstrated OMV‐mediated transfer as a novel mechanism for HGT of AMR genes in *Campylobacter*. To exclude the possibility of HGT mediated by the known mechanisms in the process of OMV‐mediated transfer, we took several measures, including (i) addition of DNase I to completely remove the external DNA in extracted OMVs; (ii) addition of DNase I during the process of OMV‐mediated transfer experiment to digest any DNA released from ruptured OMVs; (iii) use of a natural transformation‐deficient mutant strain (YZ2024) as a critical control and (iv) complete absence of live bacteria in the purified OMVs and use of a non‐conjugative plasmid (pRY112), eliminating the role of conjugation. Additionally, the donor and recipient strains used in the transfer experiments were devoid of phages as determined by whole genome sequencing. With these controls, the results convincingly showed that OMVs mediated HGT of antibiotic resistance determinants in *Campylobacter*.

**FIGURE 7:**
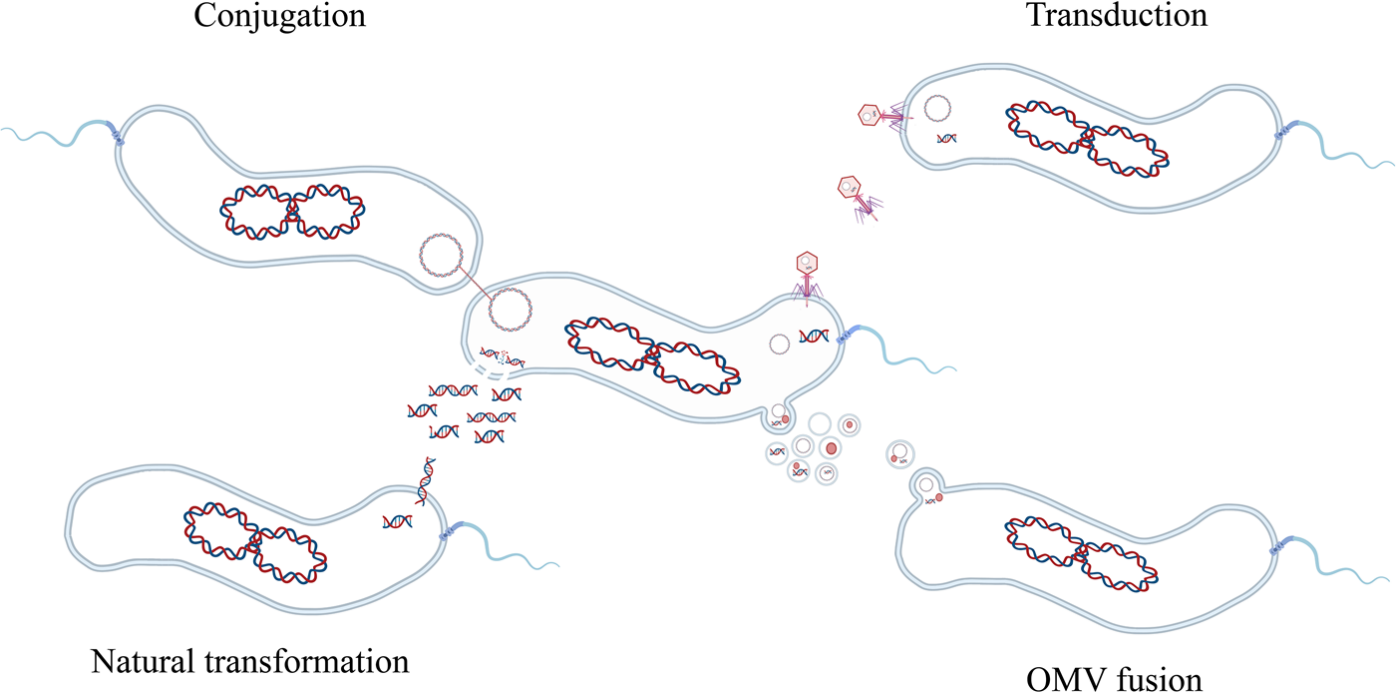
Diagram illustrating the four different mechanisms of horizontal gene transfer in *Campylobacter*, including natural transformation, conjugation, transduction and OMV fusion.

Previously, three major ways for HGT have been known in *Campylobacter*, including natural transformation, conjugation and transduction (Figure 7), each of which utilizes a distinct mechanism. Among the three mechanisms, conjugation and natural transformation are the most frequently reported and are thought to play a key role in the exchange of genetic materials in *Campylobacter* (Golz and Stingl 2021; Sheppard and Maiden 2015). Conjugation requires direct cell‐to‐cell contact and is important for transfer of plasmid‐encoded antimicrobial resistance genes (Zeng et al. 2015). However, the shuttle plasmid pRY112 used in this study lacks conjugation machinery (Yao et al. 1993), ruling out conjugation as a mechanism for its transfer. Natural transformation, another major HGT mechanism, plays an important role in generating genetic diversity in *Campylobacter* and is also widely used for genetic manipulation of the organism (Wang and Taylor 1990). Natural transformation frequencies in *Campylobacter* typically range from 10^−7^ to 10^−3^ transformants per µg DNA per recipient cell, depending on factors such as strain competence, growth phase and DNA availability (Wang and Taylor 1990; Wiesner et al. 2003; Gaasbeek et al. 2009; Jeon et al. 2009). For instance, optimal conditions can yield frequencies as high as 10^−3^ in *C. jejuni* (Wang and Taylor 1990), while less permissive conditions may result in frequencies as low as 10^−7^ (Gaasbeek et al. 2009). Notably, *Campylobacter* preferentially uptakes homologous DNA over heterologous DNA, further limiting the efficiency of plasmid transfer (Wang and Taylor 1990). Interestingly, several studies, including this one (Table 1), have failed to transfer the shuttle plasmid pRY112 into NCTC 11168 via natural transformation (Yao et al. 1993; Karlyshev and Wren 2005), although when SH89 chromosomal DNA was used as the donor, the transformation frequency reached to 10^−6^ transformants (Table 1). In contrast, both pRY112 and chromosomally encoded antibiotic resistance determinants were successfully transferred to NCTC 11168 by OMVs (Table 1), independent of natural transformation and conjugation. Based on these findings, we propose OMV‐mediated transfer as a novel HGT mechanism in *Campylobacter* (Figure 7). This mechanism operates independently of known HGT pathways and does not appear to require specialized DNA transfer machinery. It is very likely that OMVs mediates DNA transfer by direct fusion with the recipient bacterial cell envelope. This hypothesis is supported by the result from TEM observations (Figure 5), which provided ultrastructural evidence for this direct interaction. With this mode of transfer, OMVs directly “dump” their contents into the cytoplasm of recipient cells (Figure 7).

Interestingly, some of the vesiculants selected on 4 mg/L florfenicol plates exhibited MIC values below the selection concentration used on the plates (Figure 3). This suggests that these vesiculants only transiently survived the selection pressure and did not acquire permanent genetic changes conferring resistance to florfenicol at 4 mg/L. Although the exact reason for this phenomenon remains unclear, similar observations have been reported with other bacteria in other studies, where it was shown that OMVs produced by antibiotic‐resistant strains provided transient protection to susceptible strains through various mechanisms (Martinez et al. 2021; Gonzalez et al. 2016; Kim et al. 2018). For example, OMVs can confer antibiotic resistance by carrying antibiotic‐inactivating enzymes, such as β‐lactamases, which hydrolyse antibiotics. Specifically, *Klebsiella pneumoniae* carbapenemase (KPC)‐loaded OMVs have been shown to protect *Pseudomonas aeruginosa* against imipenem treatment (Zhang et al. 2023). OMVs equipped with NDM‐1 protected carbapenem‐susceptible *E. coli* from meropenem in a *Galleria mellonella* infection model (Martinez et al. 2021). Also, OMVs may protect susceptible bacteria from membrane‐active antibiotics like colistin by scavenging antibiotics from the extracellular environment, thereby preventing their entry into bacterial cells (Kulkarni et al. 2014). Additionally, OMVs may confer protection through their high content of drug‐binding proteins, such as antibiotic efflux proteins (Park et al. 2014). Consistent with these observations, we noticed that MIC values for florfenicol and ciprofloxacin increased 2‐ to 4‐fold (Figure 4) when OMVs derived from 11168‐FEcmeRAB or SH89 were added to NCTC 11168 and YZ2024 in this study, suggesting that the OMVs provided transit protection to the antibiotic‐susceptible strains. One plausible explanation is that these OMVs might be loaded with drug‐binding proteins CmeABC, a tripartite efflux complex system known to bind multiple antibiotics, including florfenicol and ciprofloxacin (Lin et al. 2002). By binding to antibiotics, the efflux proteins could reduce the antibiotic concentration available for growth inhibition. Both 11168‐FE*cmeRAB* and SH89 contained FE‐*cmeABC*, which demonstrated stronger binding to antibiotics than typical *cmeABC* (Lin et al. 2025). Thus, OMVs derived from these two strains showed a MIC‐elevating effect compared with the OMVs from 11168. Interestingly, the OMVs from SH89 had an even bigger effect on ciprofloxacin MIC than the OMVs of 11168‐*FEcmeRAB* (Figure 4), which suggests that beyond FE‐*CmeABC*, something else in SH89 also tributed to the elevated MIC of ciprofloxacin.

Although OMVs of *Campylobacter* can mediate the transfer of ARGs located on both chromosomes and plasmids, there appears to be a size‐dependent constraint on plasmid mobilization. Specifically, OMV‐mediated transfer was observed only with smaller plasmids like pRY112 (6.0 kb), whereas the 54‐kb *cfr*(C)‐harbouring plasmid remained non‐transferable by OMV despite multiple experimental attempts. Genomic characterization of *C. jejuni* SH89‐derived OMVs through next‐generation sequencing identified fragmented plasmid DNA components rather than intact pSH89 plasmid (Figure 2), strongly suggesting that the transfer failure originates from incomplete plasmid packaging within vesicles. This finding contrasts with our prior discovery that *Enterococcus* membrane vesicles (MVs) successfully transferred a 53‐kbp plasmid across species boundaries (Zhao et al. 2024). Such phylogenetic divergence in vesicular transfer efficiency implies that the impaired horizontal transfer of large plasmids may not be solely attributable to physical cargo limitations of OMVs (typically 20–300 nm in diameter), but rather involves vesicle architecture differences between Gram‐negative and Gram‐positive bacteria. For instance, the proteolipid composition of OMVs containing periplasmic DNA‐binding proteins might impose stricter size selection compared to the cytoplasmic membrane‐derived MVs of firmicutes. Further understanding of the mechanisms involved in OMV‐mediated gene transfer could provide deeper insights into the spread of antibiotic resistance and the role of OMVs in bacterial evolution.

To adapt to the antimicrobial selection pressure in animal and human medicine, *Campylobacter* has evolved the ability to acquire ARGs via HGT. Notably, many of these acquired resistance genes, such as *erm*(B) (macrolide resistance), *cfr*(C) (multidrug resistance) and *optrA* (resistance to oxazolidinones and phenicols), appear to have originated from Gram‐positive bacteria such as S*treptococcus*, *Enterococcus*, *Clostridium*, *Listeria* and *Lactobacillus* (Tang et al. 2017; Tang et al. 2020; Wang et al. 2014; Liu et al. 2020). However, the mechanism by which these Gram‐positive‐derived resistance genes are transferred to *Campylobacter* remains unclear. Based on our findings, we propose that MVs including OMVs may have facilitated the transfer of antimicrobial resistance genes from other bacterial species to *Campylobacter*. Unlike other HGT mechanisms (conjugation, transformation and transduction), MVs do not require specialized machinery for transfer of genetic materials and can mediate the transfer of resistance genes located on nonconjugative or immobile plasmids. This capability enhances the spread of resistance genes among diverse bacteria, posing a serious threat to public health (Schwechheimer and Kuehn 2015). Thus, whether OMVs serve as a potential vehicle for interphylum ARG transfer from Gram‐positive bacteria to *Campylobacter* warrants further examination.

In summary, we demonstrate in this study that OMVs produced by *Campylobacter* mediate the transfer of ARGs encoded on both plasmid and chromosome. This is a DNA transfer mechanism independent of natural transformation, conjugation and transduction, and therefore represents a new pathway for HGT in *Campylobacter*. In addition to transfer of ARGs, OMVs from antibiotic resistant *Campylobacter* also provide transient protections for antibiotic‐susceptible *Campylobacter*, benefiting the entire community for adaptation to antibiotic selection pressure. Future studies should be directed to explore the role of OMV‐mediated HGT in transferring AMR genes between *Campylobacter* and phylogenetically distinct species in the natural environments, such as the intestinal tract, where *Campylobacter* and other bacterial organisms co‐exist and exchange genetic materials.

## Conflicts of Interest

The authors declare no conflicts of interest.

## Accession Number

The draft genome sequences of the 16 vesiculants included in this study have been deposited under project number PRJNA1129909, and the genome sequence of the SH‐OMVs was deposited under accession number SRR29550849.

## Supporting information




**Supplementary Material**: jev270195‐sup‐0001‐tableS1.docx

## Data Availability

The data that support the findings of this study are available from the corresponding author upon reasonable request.
