# Supplementary material for: Identification of Outer Membrane Vesicles as a New Vehicle Mediating Antibiotic Resistance Gene Transfer in Campylobacter
Source: J Extracell Vesicles. 2025 Nov 11;14(11):e70195. doi: 10.1002/jev2.70195 (PMC12603785; doi:10.1002/jev2.70195)
Supplement: Supplementary file 1 — Supplementary Material: jev270195‐sup‐0001‐tableS1.docx [file JEV2-14-e70195-s001.docx]

Table S1. Primers used in this study

| Target genes | Primers | Primer sequences | Length (bp) |
| --- | --- | --- | --- |
| *Cj1211* | Cj1211-5F | AGACAACGCTCTATTAACGCAC | 409 |
|  | Cj1211-5R | AACAAGACGAACTCCAATTCACTGTGCTATAAGGTGTGCTATACCG |  |
| *aphA3* | aphA3-F | CGGTATAGCACACCTTATAGCACAGTGAATTGGAGTTCGTCTTGTT | 988 |
|  | aphA3-R | AAGCACAGGTAAAACCATAGCAAGCGATGAAGTGCGTAAGAACA |  |
| *Cj1211* | Cj1211-3F | TGTTCTTACGCACTTCATCGCTTGCTATGGTTTTACCTGTGCTT | 283 |
|  | Cj1211-3R | GCTAGATATTTAAATCGCACAGAGA |  |
| *CmR* | CmR-F | CATGAAAGCCGTCACAAACGGC | 517 |
|  | CmR-R | GGCAATACGCCCTGCACATATAG |  |
| *cmeABC* | SH89-cmeABC-F | TTGGACCAGTGTAAAGGCCA | 6794 |
|  | SH89-cmeABC-R | TTAACCAGTCTTGACGCGCT |  |
| *cmeB* | SH89-cmeB-F | ATTCAAGTTGGTAGCGAAGTT | 1151 |
|  | SH89-cmeB-R | AGGAATAAGTGTTGCACGGAAAT |  |
| *aphA3* | kan-519-F | GCCGGTATAAAGGGACCACC | 517 |
|  | kan-519-R | TGTCATACCACTTGTCCGCC |  |
| pRY112-P1 | P1-931-F | ACAGGACTATAAAGATACCAGGCG | 931 |
|  | P1-931-R | CTGGATGGAGGCGGATAAAGT |  |
| pRY112-P2 | P2-1059-F | AGTGCTGCAATGATACCGCG | 1059 |
|  | P2-1059-R | GCCGATTTCGGCCTATTTGGT |  |
| pRY112-P3 | P3-1002-F | AGGCAAGACCGATCCCCATT | 1002 |
|  | P3-1002-R | GCGCGTTGGCCGATTCATT |  |
| pRY112-P4 | P4-1046-F | AAGCCTGGGGTGCCTAATGA | 1046 |
|  | P4-1046-R | GAGTGAAAACTCAAGGCGGATT |  |
| pRY112-P5 | P5-902-F | ATGATGGGGGGCTAGCGAA | 902 |
|  | P5-902-R | CTCAAAATAAGCTTTGCCAGTGGT |  |
| pRY112-P6 | P6-956-F | AGCCCTTAGCGGTAAATATGCTA | 956 |
|  | P6-956-R | TCATTAGCTGTTGCTTCGCCT |  |
| pRY112-P7 | P7-1008-F | ATCCCGAAGTGCATAGCTTAGA | 1008 |
|  | P7-1008-R | CGCATTAGATGAAAACGGACAGGT |  |
| pRY112-P8 | P8-1018-F | TCCCATTCGTTCACCAAAAGCG | 1018 |
|  | P8-1018-R | GCGAACGACCTACACCGAACT |  |
